# An Invisible Early Steatosis Phenotype Defined for a Large Population-Based Cohort

**DOI:** 10.3390/biomedicines13123045

**Published:** 2025-12-11

**Authors:** Thierry Poynard, Olivier Deckmyn, Valentina Peta, Raluca Pais, Bernard Van Beers, Laurent Castera, Frederic Charlotte, Valerie Paradis, Pierre Bedossa, Dominique Valla

**Affiliations:** 1 Medical Faculty, Pitié Salpêtrière Hospital, Sorbonne University, 75013 Paris, France; 2 BioPredictive, 75013 Paris, France; olivier.deckmyn@biopredictive.com (O.D.); valentina.peta@biopredictive.com (V.P.); 3 Nutrition Department, Pitié-Salpêtrière Hospital, 75013 Paris, France; raluca.pais@aphp.fr; 4 Department of Radiology, Beaujon Hospital, 92110 Clichy, France; bernard.van.beers@aphp.fr; 5 Department of Hepatology, Hospital Beaujon, Assistance Publique-Hôpitaux de Paris, 75013 Paris, France; laurent.castera@aphp.fr (L.C.); dominique.valla@aphp.fr (D.V.); 6 Department of Anatomy Pathology, Pitié-Salpêtrière Hospital, 75013 Paris, France; frederic.charlotte@aphp.fr; 7 Department of Pathology, Beaujon Hospital, 92110 Clichy, France; valerie.paradis@aphp.fr (V.P.); pierre.bedossa@liverpath.fr (P.B.); 8 Liverpat, 75016 Paris, France; 9 UMR1149 (CRI), Inserm, Université Paris Cité, 75018 Paris, France

**Keywords:** very early steatosis, PDFF, UK-Biobank, FibroTest, SteatoTest-2, NashTest-2, FibroSure

## Abstract

**Background:** The current definition of metabolic dysfunction-associated steatotic liver disease (MASLD) relies on a classical assessment of steatosis via liver biopsy, with grades S0–S3 (5–100% fat) potentially underestimating low-grade steatosis. We propose a new, more sensitive classification based on magnetic resonance imaging–proton-density fat fraction (MRI-PDFF), splitting the existing S0 and S1 grades into three classes: new-S0, very early S1 (S1A), and later S1 (S1B). We aimed to determine whether these early S1A/S1B phenotypes differed clinically or biologically from the new-S0 grade using large population cohorts. **Methods:** We assessed the prevalence of the new MRI-PDFF—based grades in 29,252 healthy participants from the UK Biobank discovery cohort, 286 outpatients with type 2 diabetes, and in six previously published databases (*N* = 149,212) using SteatoTest-2 or a proxy. We performed a multimodal assessment of steatosis using longitudinal MRI-PDFF and liver biopsy data (*N* = 286). Models were used to adjust for phenotypes and overall mortality, controlling for age, sex, and cardiometabolic factors. **Results:** In the UK Biobank cohort, the prevalences of the new-S0, S1A, and S1B grades were 54%, 26%, and 17%, respectively. Grades S1A and new-S0 were most discriminated by triglycerides (odds ratio [OR]: 2.40, 95% confidence interval [CI]: 2.07–2.77, *p* < 0.00001) and body mass index (BMI; OR: 1.30, 95% CI: 1.27–1.33, *p* < 0.00001), and grades S1A and S1B were most discriminated by triglycerides, BMI, systolic blood pressure (SBP), and glycated hemoglobin (HbA1c). Adjusting for age, sex, SBP, BMI, HbA1c, triglycerides, and high-density lipoprotein–cholesterol) revealed significantly lower 15-year survival in the high-risk group (97.2%, 95% CI: 96.9–97.7) versus the low-risk (99.4%, 95% CI: 99.2–99.6) group (*p* < 0.00001). **Conclusions:** The early trajectory of liver steatosis is undetectable in 26% of middle-aged adults. This early steatosis phenotype differs clinically and biologically from the new-S0 grade in large population cohorts.

## 1. Introduction

The global burden of metabolic dysfunction-associated steatotic liver disease (MASLD) already affects ≈38% of adults worldwide [1,2], with global holistic and clinically clear updated definitions. MASLD is defined as steatotic liver disease (SLD) in the presence of one or more cardiometabolic risk factor(s) and the absence of harmful alcohol intake. SLD is defined as hepatic steatosis identified by imaging and or biopsy. These guidelines stated that “no suitable tests for population-based screening for SLD are currently available and the presence of steatosis per se would not necessarily prompt treatment for liver disease” [3].

Here, we try to improve the methods for constructing and validating better circulating tests for the diagnostic of early steatosis. Decision-making in healthcare relies on understanding patients’ past and current health states to predict and, ultimately, change their future course [4].

The world of hepatology took at least 20 years to recognize the true impact of the 25% uncertainty in the three major liver features of metabolic dysfunction-associated steatohepatitis (MASH): steatosis, inflammation, and fibrosis. Retrospectively, this was not due to the quality of hepatologists and pathologists but mainly due to the too small sample size of the biopsy taken as main reference.

The road to finding better methods for better tests was not easy. As several others, our group since 1979 to 2025 published 427 articles related to MASLD diagnosis, which can be retrieved by the following PubMed filter using keywords of MASLD: “(fibrosis) or (steatosis) or (cardiovascular) or (metabolic) or (hypertension) or (dyslipidaemia) and (poynard t)”.

We focused here on a limited selected key references from our group as we constructed and validated biomarkers combining only available components. According to the exponential number of publications on MASLD diagnostic tests such as the extra cellular matrix-associated diagnostics components reviewed recently [5], we also selected ten promising MASLD test components from other groups published in 2025 [6,7,8,9,10,11,12,13,14,15], which could prepare future test references.

Since 2005, the severity of the three basic features of metabolic dysfunction-associated steatotic liver disease (MASLD) has been graded using the Clinical Research Network’s (CRN) histological score [16]. Inflammatory activity is classified from A0 to A3, and fibrosis from F0 to F4. Both are expressed on a 0–100% scale, with an estimated measurement uncertainty of 20–25% for a 20 mm biopsy sample [17,18].

Graded from S0 (no fat) to S3 (severe [≥66%] fat), hepatocyte steatosis has traditionally been assessed using a 5–100% scale, an arbitrary lower threshold that poorly captures early changes in liver fat accumulation [17,19,20]. In this context, redefining low-grade steatosis with narrower, high-precision proton-density fat fraction (PDFF)–based bands may allow earlier detection and more precise monitoring. Indeed, PDFF offers tenfold greater accuracy and reproducibility than biopsy, making it suitable for identifying early trajectories of hepatic fat with very low uncertainty [21].

In a cohort of 728 patients with biopsy-confirmed MASLD, Kim et al. found that liver biopsy tended to overestimate steatosis grades compared to PDFF. Their findings suggested the following cutoffs for steatosis grading according to PDFF: S0 < 6.4%, S1 ≥ 6.4–17.4%, S2 ≥ 17.4–22.1%, and S3 ≥ 22.1% [18]. They highlight the need for more sensitive classification systems that can capture the earliest stages of liver fat accumulation.

Additionally, a reference method with only 2% uncertainty—ten times more precise than the CRN score—would better support the development of circulating noninvasive tests (NITs) for MASLD. In a recent randomized trial with 60 patients per arm, lubiprostone reduced hepatic steatosis versus placebo after 48 weeks, with a ~2% greater decrease in PDFF (−4.2% vs. −2.3%), demonstrating the clinical utility of such high-precision measurements [18,22].

The last gap in the current definition of steatosis grades is its mandatory presence for defining MASLD. This definition did not take into account both the uncertainty of biopsy but also the temporal variability such as the burning out associated with fibrosis progression [23]. A 60-year-old patient with obesity and type-2 diabetes, classified at biopsy with F3 fibrosis stage, an inflammatory activity grade A2, and a steatosis grade S0 < 5%, without any other cause of steatosis, should be included and treated as MASLD in real-life practice.

We hypothesized that further subdividing the early steatosis spectrum would identify a previously “invisible” subpopulation with distinct clinical characteristics and prognostic relevance. Therefore, we proposed splitting the standard S0 grade into two categories: new-S0, representing truly minimal steatosis, and S1A, representing very early steatosis. The traditional S1 grade becomes S1B, while the S2 and S3 grades remain unchanged.

To prove this novelty, we included large population-based cohorts and aimed to determine whether individuals with grade S1A steatosis differ clinically and biologically from those with new-S0 grade stenosis, and whether this early phenotype has prognostic value.

## 2. Materials and Methods

The rational choice for the new definition of very early steatosis was to take 3.2% the median of previous proposed definition using PDFF 6.4% and to validate this choice by demonstrating a phenotype in comparison with participants < 3.2% and those ≥ 6.4%.

The choice of the multi-cohort approach seemed appropriate to increase the identification of a real phenotype in various contexts of use as well as the statistical power.

### 2.1. Study Design

To enhance the precision of early MASLD detection, we propose splitting the traditional S0 grade into two sub-grades: new-S0 (no steatosis), defined as a PDFF of <3.2%, and S1A (very early steatosis), defined as a PDFF of ≥3.2–6.4%. The traditional S1 grade (early steatosis, PDFF of ≥6.4–17.4%) becomes S1B, while the S2 (moderate steatosis, PDFF of ≥17.4–22.1%) and S3 (severe steatosis, PDFF of ≥22.1%) grades remain unchanged. Adding the S1A grade should increase classification granularity and test sensitivity, enabling rigorous evaluation of early-stage therapies.

We first examined associations between the new stages (new-S0, S1A, and S1B) and three holistic MASLD traits—obesity, type 2 diabetes mellitus [T2DM], and systolic blood pressure (SBP)—in two prospective datasets: the ostensibly healthy participants in the large UK Biobank [24,25] and in the subset of outpatients with T2DM in the QUID-NASH project [26] (Table 1). Then, we conducted a post hoc analysis of the prevalence of steatosis grades across five prospective MASLD datasets (Table 2) [27,28,29,30]. Finally, we explored whether the S1A grade differs clinically and biologically from the new-S0 grade and the traditional S1B grade in these various contexts.

### 2.2. Outcomes

Among the concepts, which were better described in the past decade, are the temporal trajectories of the three features defining MASLD [31]. The consensus is that these features, if they occurred, the first step is steatosis, followed by inflammatory activity (MASH), and then fibrosis. In the worst-scenario, almost always in patients with significant fibrosis, severe complications occurred such as hemorrhage, encephalopathy, or primary liver cancer. Therefore, the early diagnostic of steatosis occurrence and progression is a major challenge for MASLD surveillance and treatment.

The primary outcome was to determine whether the new S1A grade identifies participants at higher cardiometabolic risk in the UK Biobank discovery cohort. Risk was scored from 0 to 5 using the five criteria specified in the new nomenclature [5] (Table 1): hypertension (SBP ≥ 130 mmHg), obesity (body mass index [BMI] ≥ 25 kg/m^2^ or ≥ 23 kg/m^2^ for those with Asian ancestry), high triglyceride levels (≥1.70 mmol/L [≥150 mg/dL]), low high-density lipoprotein–cholesterol (HDL-C; ≤1.3 mmol/L [≤50 mg/dL] for females; ≤1.0 mmol/L [≤40 mg/dL] for males), and T2DM (glycated hemoglobin [HbA1c] ≥ 5.7% [≥39 mmol/L]).

The second outcome was to determine whether 15-year all-cause mortality differed significantly between the S1A grade and the new-S0 or S1B grades in the UK Biobank discovery cohort. A Cox proportional-hazards analysis (hereafter the risk model), incorporating the same five cardiometabolic traits, quantified their relative impact: participants whose predicted mortality exceeded the cohort median were classified as high risk and all others as low risk.

The third outcome was to quantify how the revised PDFF cutoffs (<3.2% and ≥3.2–6.4%) arithmetically inflate the prevalence of early-stage MASH, which was previously excluded by the <5% PDFF threshold for steatosis in the steatotic liver disease (SLD) criteria [3]. Given that MASLD already affects ≈38% of adults worldwide, we recalculated the UK age-standardized rate per 100,000 in the UK Biobank discovery cohort, adjusting it by the percentage increase caused by the new early steatosis category. Finally, as a sensitivity analysis, we applied the cardiometabolic liver phenotypes described by Raverdy et al. (MASLD clusters) [2] to two prospective datasets with complete cluster data.

### 2.3. Study Population

**Figure 1:**
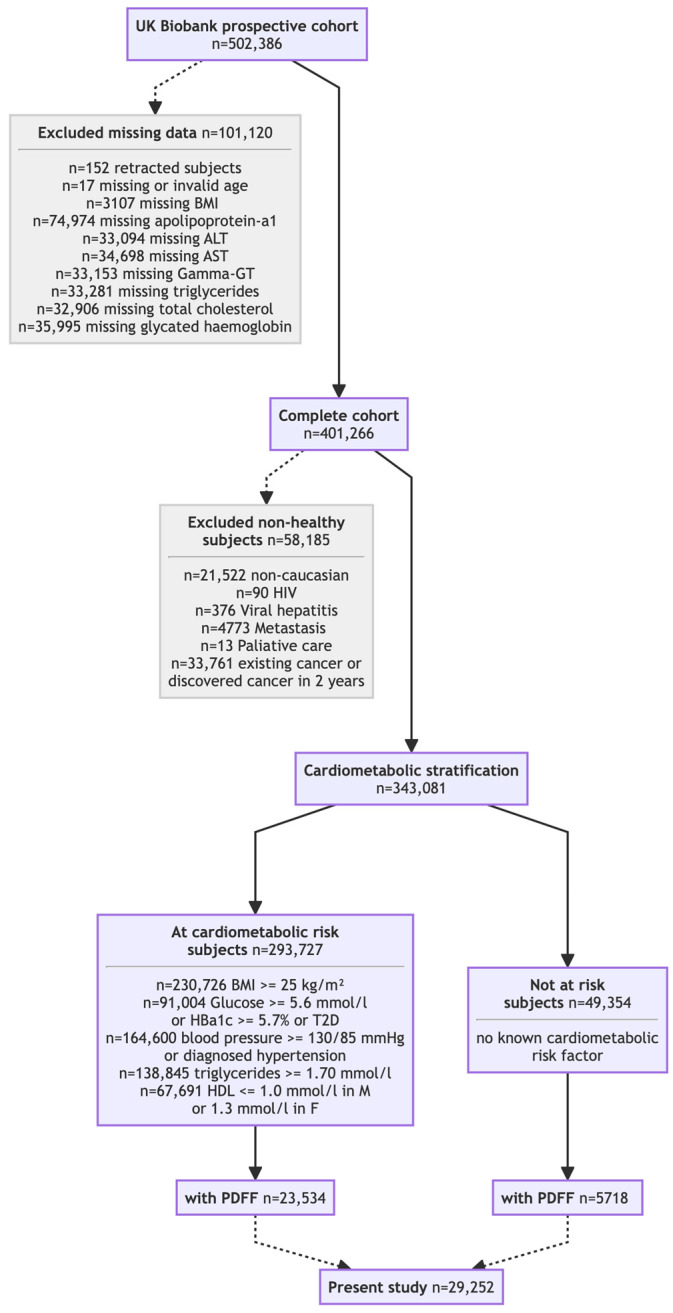
Flow chart of the UK Biobank cohort included subset.

The impact of the new steatosis grading system was assessed across seven cohorts. Two core cohorts (Table 1) underwent prospective PDFF: the UK Biobank discovery cohort (*N* = 29,252; Figure 1) [24,25] and the QUID-NASH clinic cohort (*N* = 286) [26]. Unlike the population-based UK Biobank discovery cohort, the QUID-NASH clinic cohort comprises outpatients with T2DM, abnormal liver test results, and a very high risk of MASLD at a tertiary center (Paris, France). Therefore, the UK Biobank discovery cohort enabled the prevalence of the new-S0, S1A, and S1B grades to be estimated in ostensibly healthy adults [24,25]. Analyses were restricted to participants classified as White to reduce confounding due to ancestry. Five cohorts described in four publications [27,28,29,30] without PDFF data (*N* = 149,212, Table 2) were re-graded using NITs. SteatoTest-2, embedded in FibroSure^®^ Plus (LabCorp, Burlington VA, USA) and Nash-FibroTest (BioPredictive, Paris, France)—henceforth referred to as NashTest/FibroSure—was the primary test; when unavailable, a validated SteatoTest-2 proxy was used. The *Caisse Primaire d’Assurance Maladie* (CPAM) cohort represents the general French adult population [27]. The USA-FibroSure and France-FibroTest cohorts represent large laboratory series of subjects at risk of fibrosis [28]; the France-FibroTest cohort also includes a subset with prospective biopsies (*N* = 1081) [29]. The peginterferon alfa (PEG-IFN)–Ribavirin cohort (*N* = 1428) represents a post hoc analysis of a chronic hepatitis C trial [30]. Male and female adults with prospectively collected anonymized data were included, enabling the NIT-based assessment of the three key features of MASLD: steatosis grade, inflammatory activity grade, and fibrosis stage.

In a subset of the UK Biobank discovery cohort (~10%), imaging and biochemistry were performed during follow-up at a median of four years after inclusion [24,25].

### 2.4. Data Sources and Study Tools

Steatosis was assessed based on PDFF (almost perfect reference) and two validated blood-based NITs: serum triglycerides and the composite NashTest/FibroSure. Histological assessment used both the standard CRN score [16] and our proposed refined version, which splits the S0 and S1 grades into new-S0, S1A, and S1B grades but retains the traditional S2 and S3 grades. Consistent with the new SLD classification, we also assessed the prevalence of overweight, T2DM, and SBP, as well as age, sex, and alcohol intake as potential confounders [1,2,3]. The examinations were performed according to the manufacturer’s recommendations: the UK Biobank for the PDFF [24,25] and BioPredictive (Paris, France) for NashTest/FibroSure. The standard PDFF distribution cutoffs from Kim et al. followed the recently established CRN steatosis grading system [18]. The proposed new steatosis grading system was applied as described in Section 2.1. The histological grading of hepatocyte steatosis followed the cutoffs in the standard CRN steatosis grading system: S0 = 0–<5%, S1 ≥ 5–<33%, S2 ≥ 33–<66%, and S3 ≥ 66–100% [16]. The NashTest/FibroSure components SteatoTest2, NashTest2, and FibroTest were used to assess the prevalence of CRN MASH grades and fibrosis stages in a large population.

### 2.5. Ethical Considerations

Informed consent has been obtained from all subjects as part of the seven studies already published. All procedures conformed to the Declaration of Helsinki. This study was approved by the Ethics Committee of the Faculty of Medicine at Helwan University (approval number: 11-2020, approval date: 11 February 2020). All participants in the UK Biobank provided written informed consent as part of their recruitment for that project [23,24] (Present Application 79974; date 23 November 2022). The QUID-NASH project (ClinicalTrails.gov: NCT03634098) was approved by the Research Ethics Committee (approval number: 18.021-2018-A00311-54, access date 23 November 2022) [26]. The CPAM study enrolled adults aged ≥40 years screened at two Social Security centers; all procedures adhered to the Declaration of Helsinki and were approved by the ethics board at University Hospitals Pitié-Salpêtrière, and written informed consent was obtained from all subjects [27]. The USA-FibroSure and France-FibroTest cohorts were large anonymous BioPredictive series of subjects at risk of MASLD [28]. All authors accessed the data and approved the manuscript. The FibroFrance project (ClinicalTrials.gov: NCT01927133) was funded by the National Clinical Research Program, and clinical conduct adhered to the principles outlined in the Declaration of Helsinki [29]. The PEG-IFN–Ribavirin trial, conducted at 62 centers worldwide, obtained written informed consent from all subjects and was approved by the local ethics board at each site [30].

### 2.6. Statistical Analysis

Clinical interest in a new phenotype depends on its utility for diagnosis or prognosis relative to the old phenotypes. The proposed revised SLD grading system, which introduces a new grade (S1A) representing very early steatosis between the no (S0) and early (S1B) stenosis grades, enhances granularity without altering the S2 (moderate) or S3 (severe) grades. First, univariate and multivariate models were employed to distinguish S1A from new-S0 and S1B. The models were constructed following matrix correlation assessment and adjusted for age and sex. Their discriminative performance was assessed using odds ratios (ORs) with 95% confidence intervals (CIs). Then, whether baseline S1A and S1B grading improved the median four-year prediction of incident S2–S3 steatosis versus S0 grade among participants who underwent repeat PDFF measurements was assessed.

Pancreatic and visceral fat, as measured by the PDFF, were detailed and compared between participants with S1A and S0 grades after adjustment for age in the entire cohort and separately in women and men.

Fifteen-year trajectories and survival were analyzed in participants with repeated PDFF measurements. Survival curves were compared using the Kaplan–Meier method and the log-rank test. Model risk was classified as low when the predicted 15-year multivariate risk was below the cohort median and high when it exceeded it. Overall, 15-year mortality was examined twice: first, to determine whether the baseline count of cardiometabolic factors predicted death, and second, in the longitudinal subset, to test whether four-year shifts in early steatosis grade (new-S0, S1A, and S1B) affected mortality. Multiple testing was controlled via Bonferroni adjustment, Tukey–Kramer pairwise CIs and *p*-values, and the Cochran–Armitage trend test with continuity correction. All statistical analyses were performed using NCSS (version 2024) and R (version 4.4) statistical software with standard libraries, including dplyr, Cox models, pROC, and ANCOVA.

## 3. Results

The primary finding of this study was the identification of a previously unrecognized early steatosis phenotype (S1A grade) with distinct clinical features and potential prognostic relevance.

### 3.1. Prevalence and Differences in Clinical Characteristics

Our results revealed the characteristics of the new early steatosis grade, S1A, which is distinct from the new-S0 grade, which served as the control, and from the S1B grade, the next grade. The prevalences of the three new grades were 53.6% (95% CI: 0.53–0.54%) for new-S0, 26.1% (95% CI: 25.6–26.6%) for S1A, and 16.6% (16.2–17.0%) for S1B (Table 1). The prevalence of the S0 grade, the lowest of the four grades in the Kim et al. [18] classification, was 79.7% (95% CI: 79.2–80.1%), 26.1% higher than that of the new-S0 grade.

The multivariate logistic regression and the univariate analysis of covariance (ANCOVA) identified several clinically significant characteristics that differed between the new-S0 and S1A and between S1A and S1B grades, adjusting for age, sex, BMI, SBP, HbA1c level, triglyceride level, and HDL-C level. In the multivariate comparisons, the most discriminating characteristic between S1A and new-S0 was BMI (OR: 1.30, 95% CI: 1.27–1.33, *p* < 0.00001), and the most discriminating characteristics between S1A and S1B were triglycerides (OR: 0.67, 95% CI: 0.59–0.77, *p* < 0.00001), BMI (OR: 0.85, 95% CI: 0.84–0.87, *p* < 0.00001), SBP (OR: 0.992, 95% CI: 0.989–0.997, *p* = 0.001), and HbA1c (OR: 0.98, 95% CI: 0.97–0.99, *p* = 0.001).

**Figure 2:**
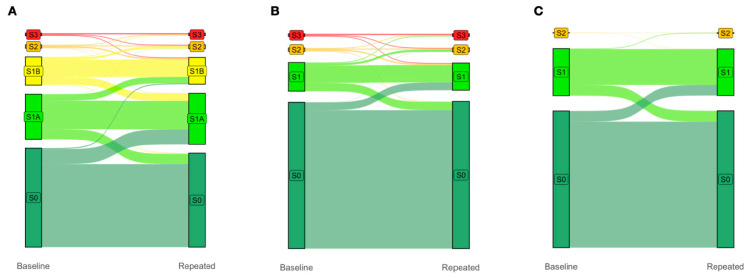
The earlier S1A and S1B grades improve the four-year prediction of clinically significant S2/S3 vs. S0 (control; *N* = 2012 with paired PDFF, *p* < 0.0001). Change in gradings under the (**A**) proposed five-grade PDFF-based classification, (**B**) four-grade PDFF-based classification proposed by Kim et al., and (**C**) four-grade CRN classification between baseline and four-year repeated PDFF measurements. The columns display the proportions of subjects with each grade at baseline (left column) and four years later (right column). The colored flow linking the two columns shows the proportion of participants who moved from one grade to another over a median of four years. Note that the trajectories between S0, S1A, and S1B are invisible in (**B**), and between S0, S1A, S1B, S2, and S3 in (**C**). Abbreviations: PDFF, proton-density fat fraction; S0, new absence of steatosis; S1A, very early steatosis; S1B, very early steatosis.

Finally, due to the greater granularity, the inclusion of the earlier S1A and S1B grades (Figure 2A) in the proposed five-grade PDFF-based classification system improved the four-year prediction of clinically significant S2–S3 vs. S0 (control; *n* = 2012 with paired PDFF, *p* < 0.0001) compared to both the traditional four-grade PDFF-based classification system proposed by Kim et al. [18] (Figure 2B) and the standard four-grade CRN classification system (Figure 2C), neither being able to identify the progression of steatosis from grades S0 to S1A to S1B then to S2/S3 (Figure 2A). Notably, in this population-based analysis, the highest flows were from the new-S0 to S1A grades (Figure 2A).

### 3.2. Differences in Prognostic Ability

**Figure 3:**
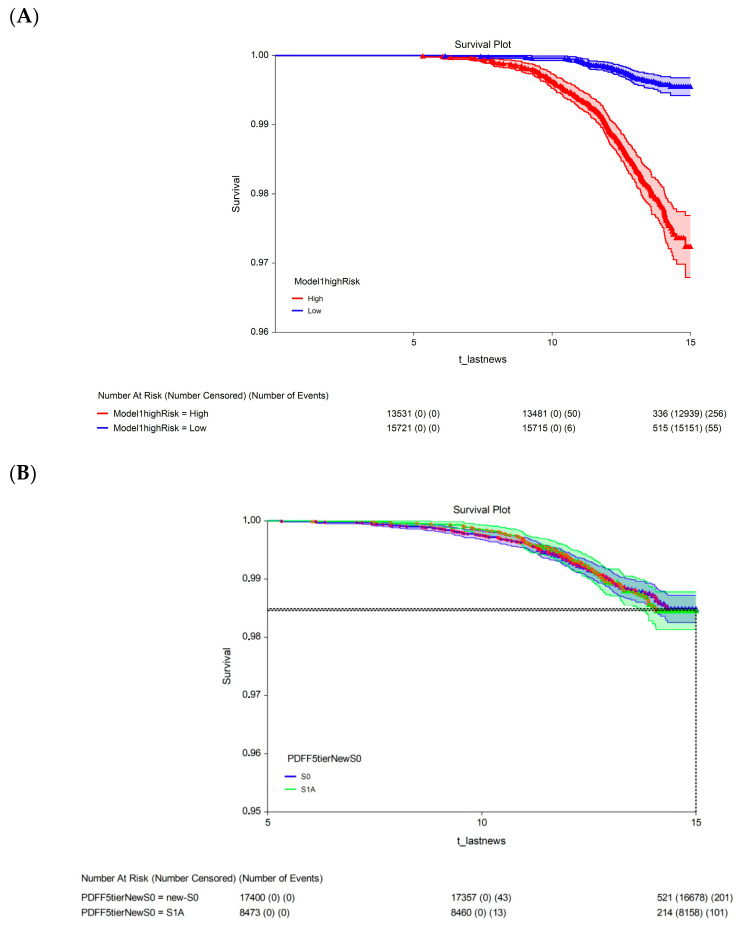

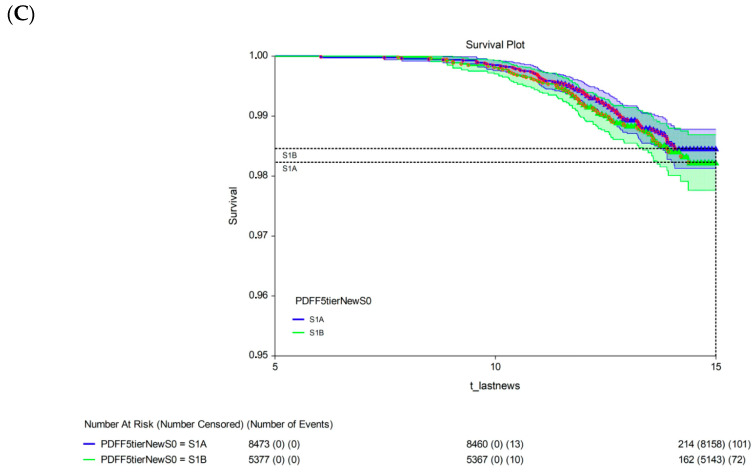
Fifteen-year all-survival model. (**A**) Survival of participants in the low- and high-risk groups after Cox multivariate adjustment. (**B**) Comparison of survival between S1A and S0 without adjustments. (**C**) Comparison of survival between S1A and S1B without adjustments. Abbreviations: PDFF, proton-density fat fraction; S0, new absence of steatosis; S1A, very early steatosis; S1B, very early steatosis.

The 15-year survival rate differed significantly between participants with a 15-year multivariate risk above the median in the cohort (high-risk; 97.3%, 95% CI: 96.9–97.7) and those with a 15-year multivariate risk below the median in the cohort (low-risk; 99.4%, 95% CI: 99.2–99.6; *p* < 0.00001; Figure 3A). The univariate analysis revealed no significant difference in 15-year survival between participants with S1A grade (98.5%, 95% CI: 98.1–98.8) and those with the new-S0 (98.5%, 95% CI: 98.3–98.7; *p* = 0.720, log-rank test; Figure 3B) or S1B (98.2%, 95% CI: 97.8–98.7; *p* = 0.430, log-rank test; Figure 3C) grades.

**Figure 4:**
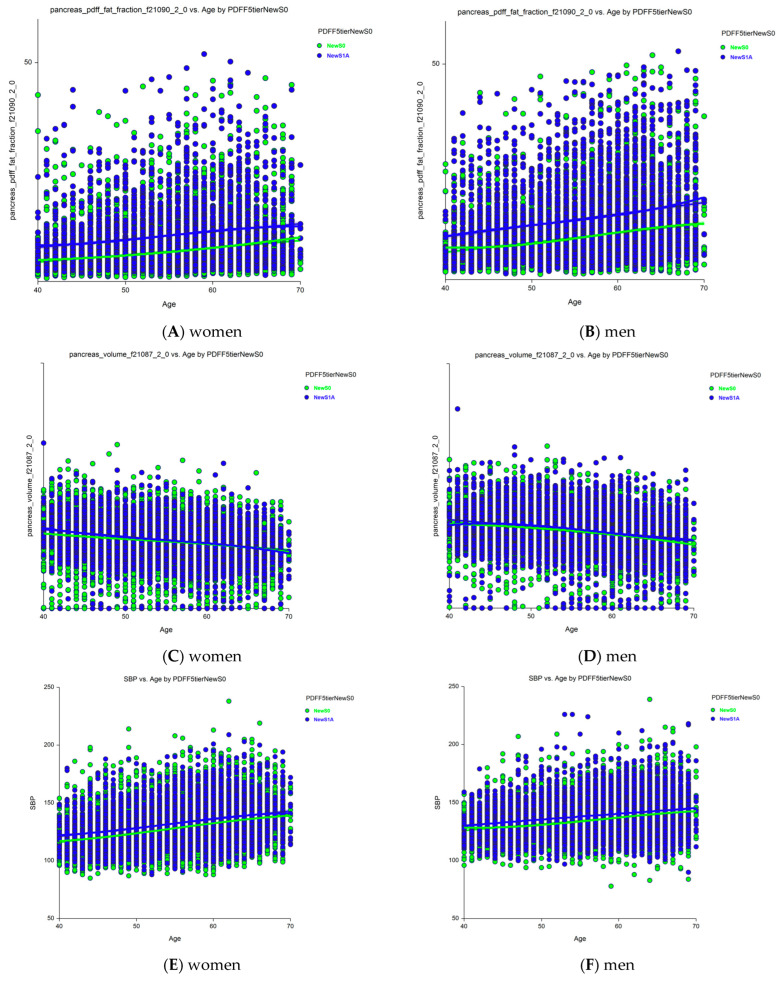
Changes in pancreatic PDFF and volume with age in those with grades S1A versus new-S0 (control). (**A**) Pancreatic PDFF were much higher in S1A vs. new-S0 and increases with age in women. (**B**) Pancreatic PDFF were much higher in S1A vs. new-S0 and increases with age in men. (**C**) Pancreas volume was slightly higher in S1A vs. new-S0 before 50 years and decreased with age. (**D**) Pancreas volume was slightly higher in S1A vs. new-S0 and decreased with age in men. All differences were significant *p* < 0.0001. (**E**) SBP was higher in grade S1A vs. new-S0 in women. (**F**) SBP was higher in grade S1A vs. new-S0 in men. Abbreviations: PDFF, proton-density fat fraction; S0, new absence of steatosis; S1A, very early steatosis; S1B, very early steatosis.

Sixteen holistic candidates for severity markers of S1A vs. new-S0 grades were analyzed separately in women (File S1) and men (File S2) using ANCOVA, adjusting for age (*n* = 32 comparisons). The most notable results were the increases in pancreatic PDFF with age in women (Figure 4A) and men (Figure 4B), and an unexpected decrease in pancreas volume with age, which was still higher in those with early steatosis (S1A grade) than in those with no stenosis (new-S0 grade) among women (Figure 4C) and men (Figure 4D). Age was associated with all characteristics, except HbA1c level and total adipose tissue volume in both women and men. The liver and pancreas volumes decreased with age, whereas the pancreatic PDFF increased with age, unlike the hepatic PDFF.

Thirty of the 32 characteristics were significantly elevated in those with S1A grade compared to those with the new-S0 grade (*p* < 0.0001): SBP, BMI, liver volume, visceral fat volume, subcutaneous fat volume, visceral adipose tissue volume, total trunk fat volume, total adipose tissue volume, pancreas volume, hepatic iron, and pancreatic PDFF. Only HbA1c levels did not differ significantly between those with the S1A and new-S0 grades in both women and men. Pancreatic iron was normal in women and only marginally elevated in men (*p* = 0.035). SBP was significantly higher in those with S1A grade compared to those with new-S0 grade (*p* < 0.00001, Figure 4) and increased with age in both women (Figure 4A, *p* < 0.00001) and men (Figure 4B, *p* < 0.00001).

As in the UK Biobank subset, PDFF was also assessed in the pancreas and visceral tissue together with 32 combinations of holistic factors. We compared the characteristics of participants with a very early steatosis grade S1A with those having the newS0 definition observed in hepatocytes, also stratified by age and sex, and the association between very early steatosis with holistic characteristics, which was the fourth outcome (post hoc).

The last and fifth outcome was to assess the robustness of the new early steatosis grades by comparing all combinations of steatotic liver disease characteristics between S1A vs. new-S0 grades, separately in women and men (File S3), using ANCOVA, adjusted for age (*N* = 32 comparisons).

### 3.3. Impact of the S1A Grade on the Definition of SLD

At baseline, the prevalence of liver steatosis among participants in the UK Biobank was 34% higher with PDFF-based classifications than with the standard histological classification, which exhibits approximately 25% diagnostic uncertainty for a 20 mm biopsy sample [17,19]. MASLD, the most common chronic liver disease worldwide, has a global prevalence of 38% among adults. However, based on our results, the true prevalence of MASLD could be at least 13% higher (38% × 34% = 13%), yielding a prevalence of at least 51% [1]. As expected, participants in the UK Biobank population-based cohort with the S0, S1A, and S1B grades (*N* = 1498, 173 with repeated PDFF) all matched the “control phenotype” in the MASLD cluster analysis (File S3). All outpatients with T2DM in the QUID-NASH cohort (*n* = 314) were classified into the “cardiometabolic SLD” MASLD cluster.

The *Caisse Primaire Assurance Maladie* (CPAM) cohort is representative of the general French adult population [27]. The USA-FibroSure and France-FibroTest cohorts represent large laboratory series of subjects at risk of fibrosis [28], which are the appropriate cohorts for the context of surveillance in patients at risk of MASLD with balanced fibrosis stage spectra; the France-FibroTest cohort also includes a subset with prospective biopsies with more severe spectrum (*N* = 1081) [29]. The peginterferon alfa (PEG-IFN)–Ribavirin cohort (*N* = 1428) represents a post hoc analysis of a chronic hepatitis C trial. This large cohort allowed the assessment of the role genotype-3 in the occurrence of steatosis, as well as the interest of SteatoTest as a noninvasive biomarker of steatosis surveillance, simultaneously with fibrosis staged by FibroTest [30].

## 4. Discussion

Our findings did not fit within the most current MASLD/MASH literature but are in line with those highlighting the impact of uncertainty [26], granularity [18,22,32], and temporal variabilities among steatosis, activity inflammation (MASH), and fibrosis [22,23,31]. Since 2005, the Nash-FibroTest, the most widely used patented circulating test in the USA for SLD, has remained the only blood test that assesses the grades of steatosis (SteatoTest-2) and MASH (NashTest-2), as well as the stages of fibrosis (FibroTest) using a single blood sample [26,27,28,29]. Our findings are also in line with the holistic associations between early liver features and the occurrence of diseases in several other organs [2,4,5,6,7,9,10,24,33,34,35,36,37]. The S1A grade proposed in our study, which is associated with higher triglyceride levels and BMI than the S0 grade, confirms observations from the third National Health and Nutrition Examination Survey and the National Death Index for an index combining triglycerides and BMI in individuals without advanced fibrosis [33].

Thanks to the UK Biobank, our findings indicate that the prevalence of hepatic steatosis was 46.4% among participants in the UK Biobank, substantially higher than the previously reported 21.3%. This difference is due to our identification of a newly defined earliest stage of steatosis (grade S1A), which was present in 26.1% of the participants (95% CI: 25.6–26.6%, Table 1).

The prevalence of participants without steatosis, using the new PDFF cutoff of <3.2% (grade new-S0), was 53.6%. These participants were previously classified as having no steatosis (grade S0) using the less sensitive PDFF cutoff of <6.4%, which is more useful for severe contexts, such as outpatients with T2DM [26].

These findings highlight the complementary roles of imaging and blood tests in improving the diagnosis of liver diseases. In 2005, the priority for patients with SLD was diagnosing severe fibrosis stages and MASH grades [17]. More recently, a phase 3 randomized controlled trial assessing resmetirom highlighted the need to increase the granularity of the scoring system for early indicators, which permitted the first approval for treating fibrosis stages F2 to F3 [38]. Unlike fibrosis, steatosis is not an optimal candidate for predicting liver-related mortality. Steatosis is the earliest indicator of MASLD [1,2,3,20]. More importantly, it is the first feature quantified using the PDFF, a near-perfect indicator [18,22], with an uncertainty tenfold lower than the standard 20 mm liver biopsy [17,21]. If confirmed through independent validation, the S1A grade could serve as a clinical target for efficient screening using NITs and early intervention using new treatments. NITs validated for producing accurate early steatosis grades would improve the surveillance and accelerate the approval of new drugs for MASLD.

### 4.1. Clinical Relevance of S1A: First Outcome

The traditional steatosis scoring system, which ranges from 5% to 100%, is no longer justified. Instead, like other major liver features such as inflammation and fibrosis, steatosis should be scored from 0% to 100%. Steatosis is considered the earliest manifestation of MASLD, although the debates over MASH and fibrosis remain stimulating [31], primarily due to the lack of a near-perfect reference indicator for inflammatory activity such as PDFF for steatosis.

Our findings strongly suggest that early steatosis (grade S1A, defined as a PDFF of 3.2–6.4%) represents an independent intermediate stage of steatosis with a distinct phenotype profile [5,6]. The S1A grade lies between the newly defined new-S0 (PDFF of ≤3.2%) and S1B (PDFF of ≥6.4–16.4%) grades. After adjusting for confounders, participants classified as S1A had significantly higher triglyceride levels and a greater prevalence of overweight than those classified as new-S0.

We confirmed the clinical relevance of the S1A grade, with 48/56 (86%) of the characteristics showing highly significant differences from the two adjacent grades (new-S0 and S1B). The S1A grade represents a stage characterized by increased SBP and various forms of steatosis not present in the new-S0 grade, including hepatic, pancreatic, subcutaneous, total trunk, and adipose tissue steatosis, all of which are associated with age but generally similar in women and men. One notable finding was the lack of a significant association between HbA1c levels and the S1A grade, regardless of age or sex (File S1).

As expected, compared to participants with grade S1B, those with grade S1A had lower triglyceride levels and a lower prevalence of overweight. Moreover, unlike grade S1A, grade S1B was associated with two major risk factors: a higher SBP, a key cardiovascular risk marker, and an elevated HbA1c, a critical indicator of T2DM and metabolic risk.

A recent UK Biobank study confirmed independent associations between the hepatic PDFF, visceral adipose tissue, and cardiac structure and function [9], suggesting that different trajectories of cardiovascular events could be identified according to earlier stages of visceral steatosis, as observed in our study for liver steatosis.

In this population of apparently healthy adults, it was expected that, at four years, a small proportion of subjects with S1B progress to severe steatosis. In such a population at low risk of MASLD, the interest of PDFF precision was to assess, in participants with less than 3.2% steatosis, the already significant proportion of S0 individuals who progress to S1A within four years. As no assessment of inflammatory activity (such as MASH grades using NashTest), it was not possible to confirm this rational hypothesis. The classification of steatosis proposed by the standard four-grade CRN or by Kim et al. [16,18] did not identify such very early flow, which could be a first warning (Figure 2).

The greater granularity of the proposed five-grade PDFF-based classification system enabled us to confirm the clinical relevance of the new grades S1A and S1B at baseline, which predicted an increased risk of clinically significant steatosis (grades S2 and S3) at four years. The absence of steatosis (grade S0) was better defined, without changes performed for the more severe grades, S2 and S3.

Improved surveillance of individuals at risk of MASLD is necessary, along with the validation of cost-effective early treatments. NITs validated for grading early steatosis will improve the quality of surveillance and accelerate the approval of new anti-steatosis and anti-fibrosis drugs. As shown in Figure 1, such NITs should detect the progression of stenosis from the early stage to clinically significant stages (grades S2 and S3).

### 4.2. Overall Mortality of Early Steatosis: Second Outcome

Analysis of 15-year survival data revealed a significant difference in mortality associated with the early steatosis grades. After adjusting for confounders, participants classified as low risk had a significantly lower 15-year survival rate than those classified as high risk. Thanks to the power afforded by the UK Biobank and the performance of the PDFF, there was a modest but significant 2.2% difference in survival. The participants in the UK Biobank were ostensibly healthy. Those who died before the five-year follow-up were excluded from the survival analysis, which could have contributed to the high 15-year survival (99.4%, 95% CI: 99.2–99.6) among the participants classified as low risk. Finally, as stated in Section 4.5 (Limitations), we acknowledge that the higher mortality of participants classified as grade S1A requires external validation.

### 4.3. Redefining Disease Prevalence: Third Outcome

Redefining the absence of steatosis through new criteria substantially increases the “visible” prevalence of MASH, MASLD, and metabolic dysfunction- and alcohol-associated liver disease (MetALD). If our results are independently validated, epidemiological and cost-effectiveness studies in the general population should be conducted, including MASLD features, as well as those of MASH and increased alcohol intake (MetALD) [3].

The performance of new NITs calibrated for these contexts should be evaluated for both surveillance and the validation of new treatments. Moreover, in the general population, the four-grade histological CRN classification system for steatosis is not clinically useful for grades S2 or S3. When defined by the PDFF, these grades are too unbalanced for this type of use. Our analysis of PDFF data from the UK Biobank produced several results across the three outcomes that could improve the clinical management of individuals at risk of MASLD.

### 4.4. Future Directions

Therefore, additional studies in younger, multiethnic, and socioeconomically diverse populations are needed. Although adjustments were made for key cardiometabolic confounders, residual confounding cannot be excluded. Therefore, external validation studies are needed, particularly for the prognostic performance at 15 years for all-cause mortality and mortality attributed to cardiovascular and liver-related causes [34,35,36,37]. The differences observed between pancreas and liver volumes and PDFF values merit further prospective investigation to explore potential associations with inflammation and fibrosis.

### 4.5. Our Study Has Several Limitations

Firstly, despite the prospective design of the seven cohorts included, the analyses were post hoc. Differences in medical interventions across steatosis stages could impact outcomes and interpretation. Trials of low-cost interventions without side effects are obviously needed in the context of controlling very early steatosis progression.

Secondly, PDFF is costly to determine and not widely available. Here, we used it not for population screening but as the best reference to validate the prevalence of early steatosis grades, which should permit the construction of widely available NITs with higher diagnostic and prognostic performances.

Thirdly, the justifications for cutoffs are often disputable for liver features, given the uncertainty of small biopsy samples as the standard reference [17,18,19,20]. Here, the redefinition of the PDFF-based cutoffs was supported by four main arguments: (i) the need to define hepatic steatosis using categories such as “normal,” “not clinically significant”, “moderate”, and “severe” for fibrosis or inflammation; (ii) PDFF has at tenfold greater accuracy than the biopsy-based CRN reference classification [18], justifying its use in the development of more granular NITs, which has permitted validation of the efficacy of resmetirom for MASLD fibrosis [38], and the efficacy of lubiprostone for MASLD steatosis [22]; (iii) the trajectories would also be tenfold more accurate than those based on histological reference; and (iv) PDFF-validated NITs will permit validation in large context-of-use populations, including general populations.

Fourthly, while PDFF estimates the volume fraction of liver lipids, the non-hepatocyte cell fraction may confound the analysis, as histological assessment measures the proportion of macroscopically fatty hepatocytes. Our results reinforce the importance of understanding the non-hepatocyte cell fraction, as this could be lower in the earlier steatosis grade S1A [39].

Fifthly, each examined cohort had specific limitations. The UK Biobank is not a nationally representative cohort, as the participants are predominantly White, middle-aged, healthier, and socioeconomically advantaged, with self-reported lifestyle data (e.g., alcohol intake and diet) [24,25]. SLD/MASLD is defined as SLD with daily alcohol use <20 g in females and <30 g in males combined with the presence of at least one metabolic risk factor detailed in Table 1 and Table 2 for each cohort.

MetALD was defined as the presence of SLD with daily alcohol use between 20 and 50 g for females and between 30 and 60 g for males, combined with at least one of the metabolic disturbances described above. ALD was defined as SLD with daily alcohol use of >50 g for females and >60 g for males without the requirement of having any metabolic risk factors. Objective alcohol biomarkers, such as phosphatidylethanol, quantify alcohol use and should help identify SLD subcategories alongside clinical history, reducing diagnostic misclassification of SLD/MASLD [40]. Only ~5% had their PDFF determined using the latest generation of MRI scanners.

Also, such study require an external validation, in particular for overall mortality.

We excluded 21,522 individuals identified as non-White to reduce potential confounding due to ethnicity (Figure 1). While we report survival outcomes across steatosis stages, we do not address the quality of life of surviving participants. Including patient-reported outcomes or functional health measures would provide a better comprehensive understanding of the long-term impact of early and progressive steatosis, but the follow-up should be longer.

The QUID-NASH dataset, though unique in combining biopsy with morphometric quantification, PDFF, SteatoTest, and several recent NITs not previously compared in outpatients with T2DM, also had limitations, including a relatively small sample size, an incomplete longitudinal follow-up, and exclusion at baseline of individuals with high alcohol intake (MetALD), limiting its applicability to the general population [26]. Only 6% of this dataset had grade S1A (Table 2). In the USA-FibroSure, France-FibroTest, and Fibro-CPAM datasets, missing data—especially on alcohol use—also limits their informativeness (Table 2).

## 5. Conclusions

Our study suggests that early-onset steatosis and early MASH were undetectable using conventional definitions. This affects a substantial proportion of ostensibly healthy individuals who could warrant attention.

## Figures and Tables

**Table 1:** Prevalence of PDFF-based steatosis grades and their main clinical and biological characteristics in two prospective datasets.

Variable	Population-Based UK Biobank Cohort [24,25]					QUID-NASH Outpatients with T2DM [26]				
Number of participants with a PDFF	29,252 (100%)					286 (100%)				
**Prevalence of steatosis, %**										
SLD Grade:	S0	S1A	S1B	S2	S3	S0	S1A	S1B	S2	S3
Proposed 5-grade PDFF-based classification (S0: <3.2, S1A: <6.4, S1B: <17.4, S2: <22.1, S3: ≥21.1)	53.6	26.1	16.6	1.9	1.8	0.1	6.0	45.0	19.0	30.0
4-grade PDFF-based classification of Kim et al. (S0: <6.4%, S1: <17.4%, S2: <22.1%, S3: ≥22.1%)	79.7		16.6	1.9	1.8	6.1		45.0	19.0	30.0
Four-grade SteatoTest/FibroSure classification (S0: <0.38, S1A: <0.57, S2: <0.69, S3: ≥0.69)	NA					9.8		28.7	40.6	21.0
Four-grade biopsy-based CRN standard	NA					3.2		21.3	54.2	21.3
**Phenotype characteristic, %**										
Sex (Female/Male)	51.0/49.0					41.0/59.0				
Ethnicity (White)	100					80.0				
Age group (<40/50/60/≥70)	1.0/29.0/48.0/22.0/0.0					20.0/20.0/27.0/26.0/7.0				
**Cardiometabolic risk factors (and cutoffs) according to recent MASLD nomenclature [5], %**										
SBP (≥130 mmHg)	53.0					56.0				
BMI (≥25 kg/m^2^)	58.0					92.0				
Triglycerides (≥1.7 mmol/L)	19.0					43.0				
HDL-C (males [≤1.0 mmol/L (≤40 mg/dL)/females [≤1.3 mmol/L (≤50 mg/dL)])	27.0/33.0					99.0/97.0				
T2DM (HbA1c ≥ 5.7% or glucose ≥ 5.6 mmol/L)	12.0					100				
Number of cardiovascular risk factors, median (range)	2 (0–5)					3 (1–5)				
MASLD clusters [19]	**Controls**					**Cardiometabolic SLD**				
Mild or high alcohol intake	22.0					0 (excluded)				
Smoking	39.0					29.0				
APOA1 (g/L) in males/females, median (IQR)	1.42 (0.29)/1.63 (0.34)					1.29 (0.23)/1.45 (0.28)				
4-grade biopsy-based CRN classification (S0: 0–<5, S1: 5–<33, S2: 33–<66, S3: ≥ 66–100)	NA					21.0/54.0/21.0/4.0				

Data are presented as percentages unless otherwise specified. Abbreviations: APOA1, apolipoprotein A1; CRN, Clinical Research Network; HbA1c, glycated hemoglobin; HDL-C, high-density lipoprotein–cholesterol; IQR, interquartile range; MASLD, metabolic dysfunction-associated steatotic liver disease; NA, not applicable; PDFF, proton-density fat fraction; SBP, systolic blood pressure; SLD, steatotic liver disease; T2DM, type-2 diabetes mellitus.

**Table 2:** The clinical and biological characteristics and prevalence of steatosis grades in five datasets without PDFF (*N* = 149,212).

Published Dataset:	FibroFrance-CPAM [27]	USA-FibroSure [28]	France-Fibrotest [28]	FibroFrance-Group [29]	Hepatitis C [30]
Context (Number of Participants)	General Population (*N* = 7399)	SLD (*N* = 72,026)	SLD (*N* = 67,278)	SLD (*N* = 1081)	Chronic Hepatitis C (*N* = 1428)
**Phenotype (cutoff), %**					
Sex (Female/Male)	45.0/55.0	54.0/46.0	41.0/59.0	38.0/62.0	34.0/66.0
Age group (<40/50/60/≥70)	43.0/33.0/13.0/11.0	18.0/29.0/21.0/32.0	39.0/28.0/15.0/18.0	21.0/30.0/22.0/27.0	15.0/59.0/11.0/15.0
Ethnicity (White/Other)	45.0/55.0	54.0/46.0	41.0/59.0	38.0/62.0	34.0/66.0
**SLD risk factor (cutoff), %**					
SBP (≥130 mmHg)	42.0	NA	NA	NA	NA
BMI (≥25 kg/m^2^ or ≥23 for Asians)	90.0	NA	NA	NA	NA
Triglycerides (<1/1–1.5/1.5–2.5/≥2.5 g/L)	43.0/33.0/13.0/11.0	18.0/29.0/21.0/32.0	39.0/28.0/15.0/18.0	21.0/30.0/22.0/27.0	15.0/59.0/11.0/15.0
HDL-C (males [≤1.0 mmol/L (≤40 mg/dL)]/females [≤1.3 mmol/L (50 mg/dL)])	8.0/9.0	NA	NA	NA	NA
T2DM (HbA1c or ≥5.7% or glucose ≥5.6 mmol/L)	26.0	51.0	35.0	40.0	23.0
Mild or high alcohol intake	48.0	NA	15.0	12.0	34.0
Smoking	18	NA	NA	NA	NA
APOA1 (g/L) in males/females, median (IQR)	1.59 (0.34)/1.78 (0.37)	1.31 (0.33)/1.49 (0.39)	1.37 (0.37)/1.55 (0.43)	1.34 (0.33)/1.47 (0.36)	1.33 (0.29)/1.56 (0.32)
**Prevalence of steatosis grades (cutoffs), %**					
4-grade SteatoTest/FibroSure classification (S0: ≤0.38, S1A: <0.57, S2: <0.69, S3: ≥0.69)	47.0/34.0/11.0/7.0	15.0/24.0/20.0/41.0	27.0/23.0/16.0/34.0	16.0/25.0/23.0/36.0	35.0/38.0/20.0/7.0

Data are presented as percentages unless otherwise specified. Abbreviations: CPAM, *Caisse Primaire d’Assurance Maladie*; LDL, low-density lipoprotein.

## Data Availability

The original contributions presented in this study are included in the article/Supplementary Material. Further inquiries can be directed to the corresponding authors).

## Supplementary Materials

The following supporting information can be downloaded at https://www.mdpi.com/article/10.3390/biomedicines13123045/s1. File S1: ANCOVA analysis S1A vs. S0 women phenotype. File S2: ANCOVA analysis S1A vs. S0 men phenotype. File S3: Medians of the six MASLD-cluster phenotype parameters and overall affectation cluster for the UK population-based cohort (*N* = 1498) at baseline with S0, S1A, or S1B.

## References

[1] Feng G., Targher G., Byrne C.D., Yilmaz Y., Wai-Sun Wong V., Adithya Lesmana C.R., Adams L.A., Boursier J., Papatheodoridis G., El-Kassas M. (2025). Global burden of metabolic dysfunction-associated steatotic liver disease, 2010 to 2021. JHEP Rep..

[2] Raverdy V., Tavaglione F., Chatelain E., Lassailly G., De Vincentis A., Vespasiani-Gentilucci U., Qadri S.F., Caiazzo R., Verkindt H., Saponaro C. (2024). Data-driven cluster analysis identifies distinct types of metabolic dysfunction-associated steatotic liver disease. Nat. Med..

[3] (2024). European Association for the Study of the Liver (EASL); European Association for the Study of Diabetes (EASD); European Association for the Study of Obesity (EASO). EASL-EASD-EASO Clinical Practice Guidelines on the management of metabolic dysfunction-associated steatotic liver disease (MASLD). J. Hepatol..

[4] Shmatko A., Jung A.W., Gaurav K., Brunak S., Mortensen L.H., Birney E., Fitzgerald T., Gerstung M. (2025). Learning the natural history of human disease with generative transformers. Nature.

[5] Karsdal M., Cox T.R., Parker A.L., Willumsen N., Sand J.M.B., Jenkins G., Hansen H.H., Oldenburger A., Geillinger-Kaestle K.E., Larsen A.T. (2025). Advances in Extracellular Matrix-Associated Diagnostics and Therapeutics. J. Clin. Med..

[6] Genovese F., Bager C., Frederiksen P., Vazquez D., Sand J.M.B., Jenkins R.G., Maher T.M., Stewart I.D., Molyneaux P.L., Fahy W.A. (2024). The fibroblast hormone Endotrophin is a biomarker of mortality in chronic diseases. Matrix Biol..

[7] Simeone P.G., Costantino S., Liani R., Tripaldi R., Di Castelnuovo A., Tartaro A., Mengozzi A., Cosentino F., Cipollone F., Consoli A. (2025). Interleukin-1β in circulating mononuclear cells predicts steatotic liver disease improvement after weight loss in subjects with obesity and prediabetes or type 2 diabetes. Cardiovasc. Diabetol..

[8] Rafaa T.A., Khudhair S.A., Mohammed Z.Y., Suleiman A.A. (2025). Genomic Exploration of Nonalcoholic Fatty Liver Disease: Insights From Gene Expression and Variation in Morbidly Obese Individuals. J. Obes..

[9] Shora H.A., El-Deen I.M., El-Lithy N.M., Abo-Elmatty D.M., Khirallah S.M. (2025). Growth differentiation factor-15: A marker for diabetic kidney disease in patients with metabolic-associated fatty liver disease. J. Diabetes Complicat..

[10] Chien S.C., Chiu H.C., Chiu Y.C., Wang R.-H., Dillera K.P.O., Lee K.-T., Tsai H.-W., Tsai Y.-S., Ou H.-Y., Cheng P.-N. (2025). Clinical Relevancies of Sarcopenic Obesity in Patients with Metabolic Dysfunction-Associated Fatty Liver Disease (MASLD). Dig. Dis. Sci..

[11] Askeland A., Rasmussen R.W., Gjela M., Frøkjær J.B., Højlund K., Mellergaard M., Handberg A. (2025). Non-invasive liver fibrosis markers are increased in obese individuals with non-alcoholic fatty liver disease and the metabolic syndrome. Sci. Rep..

[12] Jung Y., Lee S.M., Lee J., Kim Y., Lee W., Koo J.N., Oh I.H., Kang K.H., Kim B.J., Kim S.M. (2025). Metabolomic profiling reveals early biomarkers of gestational diabetes mellitus and associated hepatic steatosis. Cardiovasc. Diabetol..

[13] Wang H., Xu X., Shi L., Huang C., Sun Y., You H., Jia J., He Y.W., Kong Y. (2025). Identification of growth differentiation factor 15 as an early predictive biomarker for metabolic dysfunction-asso steatohepatitis: A nested case-control study of UK Biobank proteomic data. Diabetes Obes. Metab..

[14] Bitterer F., Kupke P., Adenugba A., Evert K., Glehr G., Riquelme P., Scheibert L., Preverin G., Böhm C., Hornung M. (2024). Soluble CD46 as a diagnostic marker of hepatic steatosis. EBioMedicine.

[15] Jara M., Norlin J., Kjær M.S., Almholt K., Bendtsen K.M., Bugianesi E., Cusi K., Galsgaard E.D., Geybels M., Gluud L.L. (2025). Modulation of metabolic, inflammatory and fibrotic pathways by semaglutide in metabolic dysfunction-associated steatohepatitis. Nat. Med..

[16] Kleiner D.E., Brunt E.M., Van Natta M., Behling C., Contos M.J., Cummings O.W., Ferrell L.D., Liu Y.-C., Torbenson M.S., Unalp-Arida A. (2005). Design and validation of a histological scoring system for nonalcoholic fatty liver disease. Hepatology.

[17] Ratziu V., Charlotte F., Heurtier A., Gombert S., Giral P., Bruckert E., Grimaldi A., Capron F., Poynard T., LIDO Study Group (2005). Sampling variability of liver biopsy in nonalcoholic fatty liver disease. Gastroenterology.

[18] Kim B.K., Bernstein N., Huang D.Q., Tamaki N., Imajo K., Yoneda M., Sutter N., Jung J., Nguyen K., Nguyen L. (2023). Clinical and histologic factors associated with discordance between steatosis grade derived from histology vs. MRI-PDFF in NAFLD. Aliment. Pharmacol. Ther..

[19] Poynard T., Deckmyn O., Pais R., Aron-Wisnewsky J., Peta V., Bedossa P., Charlotte F., Ponnaiah M., Siksik J.-M., Genser L. (2025). Three Neglected STARD Criteria Reduce the Uncertainty of the Liver Fibrosis Biomarker FibroTest-T2D in Metabolic Dysfunction-Associated Steatotic Liver Disease (MASLD). Diagnostics.

[20] Kanwal F., Neuschwander-Tetri B.A., Loomba R., Rinella M.E. (2024). Metabolic dysfunction-associated steatotic liver disease: Update and impact of new nomenclature on the American Association for the Study of Liver Diseases practice guidance on nonalcoholic fatty liver disease. Hepatology.

[21] McHugh L.C., Snyder K., Yager T.D. (2019). The effect of uncertainty in patient classification on diagnostic performance estimations. PLoS ONE.

[22] El-Kassas M., Mostafa H., Abdellatif W., Shoman S., Esmat G., Brahmania M., Liu H., Lee S.S. (2025). Lubiprostone Reduces Fat Content on MRI-PDFF in Patients with MASLD: A 48-Week Randomised Controlled Trial. Aliment. Pharmacol. Ther..

[23] Liu W.Y., Huang S., Ji H., Kim S.U., Yip T.C.-F., Wong G.L.-H., Petta S., Tsochatzis E., Nakajima A., Bugianesi E. (2025). From “Burnt-Out” to “Burning-Out”: Capturing Liver Fat Loss in Patients with Advanced Metabolic Dysfunction-Associated Steatotic Liver Disease From a Dynamic Perspective. Gastroenterology.

[24] Lv Z., Fu Y., Ma Y., Liu C., Yuan M., Gao D. (2025). Associations Between Visceral and Liver Fat and Cardiac Structure and Function: A UK Biobank Study. J. Clin. Endocrinol. Metab..

[25] Littlejohns T.J., Holliday J., Gibson L.M., Garratt S., Oesingmann N., Alfaro-Almagro F., Bell J.D., Boultwood C., Collins R., Conroy M.C. (2020). The UK Biobank imaging enhancement of 100,000 participants: rationale, data collection, management and future directions. Nat. Commun..

[26] Castera L., Laouenan C., Vallet-Pichard A., Vidal-Trécan T., Manchon P., Paradis V., Roulot D., Gault N., Boitard C., Terris B. (2023). High Prevalence of NASH and Advanced Fibrosis in Type 2 Diabetes: A Prospective Study of 330 Outpatients Undergoing Liver Biopsies for Elevated ALT, Using a Low Threshold. Diabetes Care.

[27] Poynard T., Lebray P., Ingiliz P., Varaut A., Varsat B., Ngo Y., Norha P., Munteanu M., Drane F., Messous D. (2010). Prevalence of liver fibrosis and risk factors in a general population using noninvasive biomarkers (FibroTest). BMC Gastroenterol..

[28] Poynard T., Deckmyn O., Munteanu M., Ngo Y., Drane F., Castille J.M., Housset C., Ratziu V. (2015). Awareness of the severity of liver disease re-examined using software-combined biomarkers of liver fibrosis and necroinflammatory activity. BMJ Open.

[29] Poynard T., Munteanu M., Charlotte F., Perazzo H., Ngo Y., Deckmyn O., Pais R., Merrouche W., de Ledinghen V., Mathurin P. (2018). Diagnostic performance of a new noninvasive test for nonalcoholic steatohepatitis using a simplified histological reference. Eur. J. Gastroenterol. Hepatol..

[30] Poynard T., Ratziu V., McHutchison J., Manns M., Goodman Z., Zeuzem S., Younossi Z., Albrecht J. (2003). Effect of treatment with peginterferon or interferon alfa-2b and ribavirin on steatosis in patients infected with hepatitis C. Hepatology.

[31] Ratziu V. (2017). Back to Byzance: Querelles byzantines over NASH and fibrosis. J. Hepatol..

[32] Sanyal A.J., Castera L., Wong V.W.-S. (2023). Noninvasive Assessment of Liver Fibrosis in NAFLD. Clin. Gastroenterol. Hepatol..

[33] Chen Q., Hu P., Hou X., Sun Y., Jiao M., Peng L., Dai Z., Yin X., Liu R., Li Y. (2024). Association between triglyceride-glucose related indices and mortality among individuals with non-alcoholic fatty liver disease or metabolic dysfunction-associated steatotic liver disease. Cardiovasc. Diabetol..

[34] Targher G., Valenti L., Byrne C.D. (2025). Metabolic Dysfunction-Associated Steatotic Liver Disease. N. Engl. J. Med..

[35] Chan W.-K., Petta S., Noureddin M., Goh G.B.B., Wong V.W.-S. (2024). Diagnosis and noninvasive assessment of MASLD in type 2 diabetes and obesity. Aliment. Pharmacol. Ther..

[36] Denimal D., Ponnaiah M., Jeannin A.C., Phan F., Hartemann A., Boussouar S., Charpentier E., Redheuil A., Foufelle F., Bourron O. (2024). Non-alcoholic fatty liver disease biomarkers estimate cardiovascular risk based on coronary artery calcium score in type 2 diabetes: A cross-sectional study with two independent cohorts. Cardiovasc. Diabetol..

[37] Denimal D., Ponnaiah M., Phan F., Jeannin A.-C., Redheuil A., Salem J.-E., Boussouar S., Paulstephenraj P., Laroche S., Amouyal C. (2025). Metabolic dysfunction-associated steatotic liver disease (MASLD) biomarkers and progression of lower limb arterial calcification in patients with type 2 diabetes: A prospective cohort study. Cardiovasc. Diabetol..

[38] Harrison S.A., Bedossa P., Guy C.D., Schattenberg J.M., Loomba R., Taub R., Labriola D., Moussa S.E., Neff G.W., Rinella M.E. (2024). A Phase 3, Randomized, Controlled Trial of Resmetirom in NASH with Liver Fibrosis. N. Engl. J. Med..

[39] Qadri S., Vartiainen E., Lahelma M., Porthan K., Tang A., Idilman I.S., Runge J.H., Juuti A., Penttilä A.K., Dabek J. (2024). Marked difference in liver fat measured by histology vs. magnetic resonance-proton density fat fraction: A meta-analysis. JHEP Rep..

[40] Tavaglione F., Díaz L.A., Ajmera V., Madamba E., Singh S., Bettencourt R., Richards L., Loomba R. (2025). Clinical utility of phosphatidyl ethanol to detect underreported alcohol use and enhance steatotic liver disease subclassification. J. Hepatol..

