# Supplementary material for: An Invisible Early Steatosis Phenotype Defined for a Large Population-Based Cohort
Source: Biomedicines. 2025 Dec 11;13(12):3045. doi: 10.3390/biomedicines13123045 (PMC12731107; doi:10.3390/biomedicines13123045)
Supplement: Supplementary file 1 [file biomedicines-13-03045-s001.zip › S1 File pheno ANCOVA women.pdf]

Supplementary File S1. ANCOVA S1AvsS0 Women Phenotype.pdf

Analysis of Covariance (ANCOVA) with Two Groups Report

Dataset \\Mac\Home\Desktop\NCSS\UK Biobank\export20250109.NCSS
Filter (sex = "F") AND (baseline_pdff <> Missing) AND (PDFF5tierStp = "S0";"S1A")
Response HbA1cTable1

Analysis of Variance

Source	DF	Sum of Squares	Mean Square	F-Ratio	P-Value	Significant at 5%?
Model	2	4,94838	2,47419	2,349	0,0955	No
Age	1	0,6882991	0,6882991	0,653	0,4189	No
PDFF5tierStp	1	4,609074	4,609074	4,375	0,0365	Yes
Error	13967	14713,74	1,053465			
Total(Adjusted)	13969	14718,69	1,053668			

Response BMI

Analysis of Variance

Source	DF	Sum of Squares	Mean Square	F-Ratio	P-Value	Significant at 5%?
Model	2	38124,09	19062,04	1413,794	0,0000	Yes
Age	1	517,2719	517,2719	38,365	0,0000	Yes
PDFF5tierStp	1	38123,98	38123,98	2827,580	0,0000	Yes
Error	13569	182949,5	13,4829			
Total(Adjusted)	13571	221073,6	16,29015			

Response SBP

Analysis of Variance

Source	DF	Sum of Squares	Mean Square	F-Ratio	P-Value	Significant at 5%?
Model	2	514445,2	257222,6	915,777	0,0000	Yes
Age	1	433505	433505	1543,386	0,0000	Yes
PDFF5tierStp	1	43468,55	43468,55	154,759	0,0000	Yes
Error	12853	3610139	280,8791			
Total(Adjusted)	12855	4124584	320,8545			

Response liver_volume_f21080_2_0

Analysis of Variance

Source	DF	Sum of Squares	Mean Square	F-Ratio	P-Value	Significant at 5%?
Model	2	53,11849	26,55925	743,049	0,0000	Yes
Age	1	34,10262	34,10262	954,090	0,0000	Yes
PDFF5tierStp	1	25,33282	25,33282	708,737	0,0000	Yes
Error	12525	447,6888	0,03574362			
Total(Adjusted)	12527	500,8073	0,03997823			

Response subcutaneous_fat_volume_f21086_2_0

Analysis of Variance

Source	DF	Sum of Squares	Mean Square	F-Ratio	P-Value	Significant at 5%?
Model	2	37006,83	18503,42	1555,757	0,0000	Yes
Age	1	1122,83	1122,83	94,407	0,0000	Yes
PDFF5tierStp	1	36898,29	36898,29	3102,387	0,0000	Yes
Error	12525	148966,3	11,89351			
Total(Adjusted)	12527	185973,1	14,84578			

Response pancreas_volume_f21087_2_0

Analysis of Variance

Source	DF	Sum of Squares	Mean Square	F-Ratio	P-Value	Significant at 5%?
Model	2	0,1453187	0,07265934	297,076	0,0000	Yes
Age	1	0,1446589	0,1446589	591,454	0,0000	Yes
PDFF5tierStp	1	0,005122346	0,005122346	20,943	0,0000	Yes
Error	12105	2,96066	0,0002445816			
Total(Adjusted)	12107	3,105979	0,000256544			

Response liver_iron_f21089_2_0

Analysis of Variance

Source	DF	Sum of Squares	Mean Square	F-Ratio	P-Value	Significant at 5%?
Model	2	40,66467	20,33234	419,286	0,0000	Yes
Age	1	3,123139	3,123139	64,404	0,0000	Yes
PDFF5tierStp	1	34,05961	34,05961	702,364	0,0000	Yes
Error	10075	488,565	0,0484928			
Total(Adjusted)	10077	529,2297	0,05251857			

Response visceral_fat_volume_f21085_2_0

Analysis of Variance

Source	DF	Sum of Squares	Mean Square	F-Ratio	P-Value	Significant at 5%?
Model	2	6336,008	3168,004	2590,632	0,0000	Yes
Age	1	135,7094	135,7094	110,976	0,0000	Yes
PDFF5tierStp	1	5892,451	5892,451	4818,545	0,0000	Yes
Error	12525	15316,44	1,222869			
Total(Adjusted)	12527	21652,45	1,728462			

Response pancreas_pdff_fat_fraction_f21090_2_0

Analysis of Variance

Source	DF	Sum of Squares	Mean Square	F-Ratio	P-Value	Significant at 5%?
Model	2	48453,97	24226,98	722,240	0,0000	Yes
Age	1	12663,16	12663,16	377,506	0,0000	Yes
PDFF5tierStp	1	29616,03	29616,03	882,895	0,0000	Yes
Error	9694	325177,6	33,54421			
Total(Adjusted)	9696	373631,5	38,53461			

Response pancreas_iron_f21091_2_0

Analysis of Variance

Source	DF	Sum of Squares	Mean Square	F-Ratio	P-Value	Significant at 5%?
Model	2	2,080728	1,040364	93,380	0,0000	Yes
Age	1	1,985953	1,985953	178,253	0,0000	Yes
**PDFF5tierStp**	**1**	**0,01277568**	**0,01277568**	**1,147**	**0,2843**	**No**
Error	9694	108,0029	0,01114121			
Total(Adjusted)	9696	110,0836	0,01135351			

Response abdominal_subcutaneous_adipose_tissue_volume_asat_f22408_2_0

Analysis of Variance

Source	DF	Sum of Squares	Mean Square	F-Ratio	P-Value	Significant at 5%?
Model	2	22871,48	11435,74	1592,463	0,0000	Yes
Age	1	760,7759	760,7759	105,940	0,0000	Yes
PDFF5tierStp	1	22773,67	22773,67	3171,307	0,0000	Yes
Error	12958	93053,51	7,181163			
Total(Adjusted)	12960	115925	8,944829			

Response visceral_adipose_tissue_volume_vat_f22407_2_0

Analysis of Variance

Source	DF	Sum of Squares	Mean Square	F-Ratio	P-Value	Significant at 5%?
Model	2	5621,85	2810,925	2670,107	0,0000	Yes
Age	1	92,09376	92,09376	87,480	0,0000	Yes
PDFF5tierStp	1	5288,163	5288,163	5023,244	0,0000	Yes
Error	12960	13643,49	1,052739			
Total(Adjusted)	12962	19265,34	1,486294			
