# Supplementary material for: An Invisible Early Steatosis Phenotype Defined for a Large Population-Based Cohort
Source: Biomedicines. 2025 Dec 11;13(12):3045. doi: 10.3390/biomedicines13123045 (PMC12731107; doi:10.3390/biomedicines13123045)
Supplement: Supplementary file 1 [file biomedicines-13-03045-s001.zip › S2 File pheno ANCOVA men .pdf]

Analysis of Covariance (ANCOVA) with Two Groups Report

Dataset \\Mac\Home\Desktop\NCSS\UK Biobank\export20250109.NCSS
Filter (sex = "M") AND (baseline_pdff <> Missing) AND (PDFF5tierStp = "S0";"S1A")
Response HbA1cTable1

Analysis of Variance

Source	DF	Sum of Squares	Mean Square	F-Ratio	P-Value	Significant at 5%?
Model	2	2,605397	1,302699	1,232	0,2917	No
Age	1	1,508987	1,508987	1,427	0,2323	No
**PDFF5tierStp**	**1**	**1,237756**	**1,237756**	**1,171**	**0,2793**	**No**
Error	11574	12237,83	1,057356			
Total(Adjusted)	11576	12240,44	1,057398			

Response SBP

Analysis of Variance

Source	DF	Sum of Squares	Mean Square	F-Ratio	P-Value	Significant at 5%?
Model	2	214422,2	107211,1	425,554	0,0000	Yes
Age	1	177042,2	177042,2	702,734	0,0000	Yes
PDFF5tierStp	1	28993,67	28993,67	115,085	0,0000	Yes
Error	10720	2700725	251,9333			
Total(Adjusted)	10722	2915147	271,8846			

Response BMI

Analysis of Variance

Source	DF	Sum of Squares	Mean Square	F-Ratio	P-Value	Significant at 5%?
Model	2	18726,99	9363,494	1089,578	0,0000	Yes
Age	1	61,70404	61,70404	7,180	0,0074	Yes
PDFF5tierStp	1	18726,76	18726,76	2179,129	0,0000	Yes
Error	11271	96859,48	8,593691			
Total(Adjusted)	11273	115586,5	10,25339			

Response liver_volume_f21080_2_0

Analysis of Variance

Source	DF	Sum of Squares	Mean Square	F-Ratio	P-Value	Significant at 5%?
Model	2	59,63828	29,81914	621,826	0,0000	Yes
Age	1	49,24248	49,24248	1026,867	0,0000	Yes
PDFF5tierStp	1	12,8653	12,8653	268,284	0,0000	Yes
Error	10344	496,0374	0,04795412			
Total(Adjusted)	10346	555,6757	0,05370923			

Response visceral_fat_volume_f21085_2_0

Analysis of Variance

Source	DF	Sum of Squares	Mean Square	F-Ratio	P-Value	Significant at 5%?
Model	2	11491,92	5745,958	1822,411	0,0000	Yes
Age	1	340,2634	340,2634	107,919	0,0000	Yes
PDFF5tierStp	1	10918,62	10918,62	3462,993	0,0000	Yes
Error	10344	32614,03	3,152942			
Total(Adjusted)	10346	44105,95	4,263092			

Response subcutaneous_fat_volume_f21086_2_0

Analysis of Variance

Source	DF	Sum of Squares	Mean Square	F-Ratio	P-Value	Significant at 5%?
Model	2	12244,48	6122,241	981,902	0,0000	Yes
Age	1	45,98333	45,98333	7,375	0,0066	Yes
PDFF5tierStp	1	12243,49	12243,49	1963,645	0,0000	Yes
Error	10344	64495,7	6,235083			
Total(Adjusted)	10346	76740,18	7,417377			

Response liver_volume_f21080_2_0

Analysis of Variance

Source	DF	Sum of Squares	Mean Square	F-Ratio	P-Value	Significant at 5%?
Model	2	59,63828	29,81914	621,826	0,0000	Yes
Age	1	49,24248	49,24248	1026,867	0,0000	Yes
PDFF5tierStp	1	12,8653	12,8653	268,284	0,0000	Yes
Error	10344	496,0374	0,04795412			
Total(Adjusted)	10346	555,6757	0,05370923			

Response pancreas_volume_f21087_2_0

Analysis of Variance

Source	DF	Sum of Squares	Mean Square	F-Ratio	P-Value	Significant at 5%?
Model	2	0,1808635	0,09043176	312,241	0,0000	Yes
Age	1	0,1779284	0,1779284	614,347	0,0000	Yes
PDFF5tierStp	1	0,00546649	0,00546649	18,875	0,0000	Yes
Error	10016	2,900854	0,000289622			
Total(Adjusted)	10018	3,081718	0,0003076181			

Response liver_iron_f21089_2_0

Analysis of Variance

Source	DF	Sum of Squares	Mean Square	F-Ratio	P-Value	Significant at 5%?
Model	2	25,77492	12,88746	214,758	0,0000	Yes
Age	1	2,439391	2,439391	40,650	0,0000	Yes
PDFF5tierStp	1	22,13865	22,13865	368,920	0,0000	Yes
Error	8304	498,3173	0,06000931			
Total(Adjusted)	8306	524,0922	0,06309803			

Response pancreas_iron_f21091_2_0

Analysis of Variance

Source	DF	Sum of Squares	Mean Square	F-Ratio	P-Value	Significant at 5%?
Model	2	1,243709	0,6218546	80,498	0,0000	Yes
Age	1	0,5810843	0,5810843	75,221	0,0000	Yes
PDFF5tierStp	1	0,7423345	0,7423345	96,094	0,0000	Yes
Error	8135	62,84331	0,007725053			
Total(Adjusted)	8137	64,08701	0,007876			

Response visceral_adipose_tissue_volume_vat_f22407_2_0

Analysis of Variance

Source	DF	Sum of Squares	Mean Square	F-Ratio	P-Value	Significant at 5%?
Model	2	10549,4	5274,7	1781,839	0,0000	Yes
Age	1	333,3644	333,3644	112,613	0,0000	Yes
PDFF5tierStp	1	9987,282	9987,282	3373,789	0,0000	Yes
Error	10711	31707,31	2,960257			
Total(Adjusted)	10713	42256,71	3,944433			

Response pancreas_pdff_fat_fraction_f21090_2_0

Analysis of Variance

Source	DF	Sum of Squares	Mean Square	F-Ratio	P-Value	Significant at 5%?
Model	2	65311,7	32655,85	541,373	0,0000	Yes
Age	1	25096,1	25096,1	416,046	0,0000	Yes
PDFF5tierStp	1	36054,08	36054,08	597,709	0,0000	Yes
Error	8135	490707,1	60,32048			
Total(Adjusted)	8137	556018,8	68,33216			
