# Supplementary material for: An Invisible Early Steatosis Phenotype Defined for a Large Population-Based Cohort
Source: Biomedicines. 2025 Dec 11;13(12):3045. doi: 10.3390/biomedicines13123045 (PMC12731107; doi:10.3390/biomedicines13123045)

Supplementary File S3. Medians of the six MASLD-Clusters phenotypes parameters and overall affectation Cluster for the UK population-based cohort (n=1,498) at baseline with S0, or S1A or S1B. This cluster fitted with "Controls phenotype"

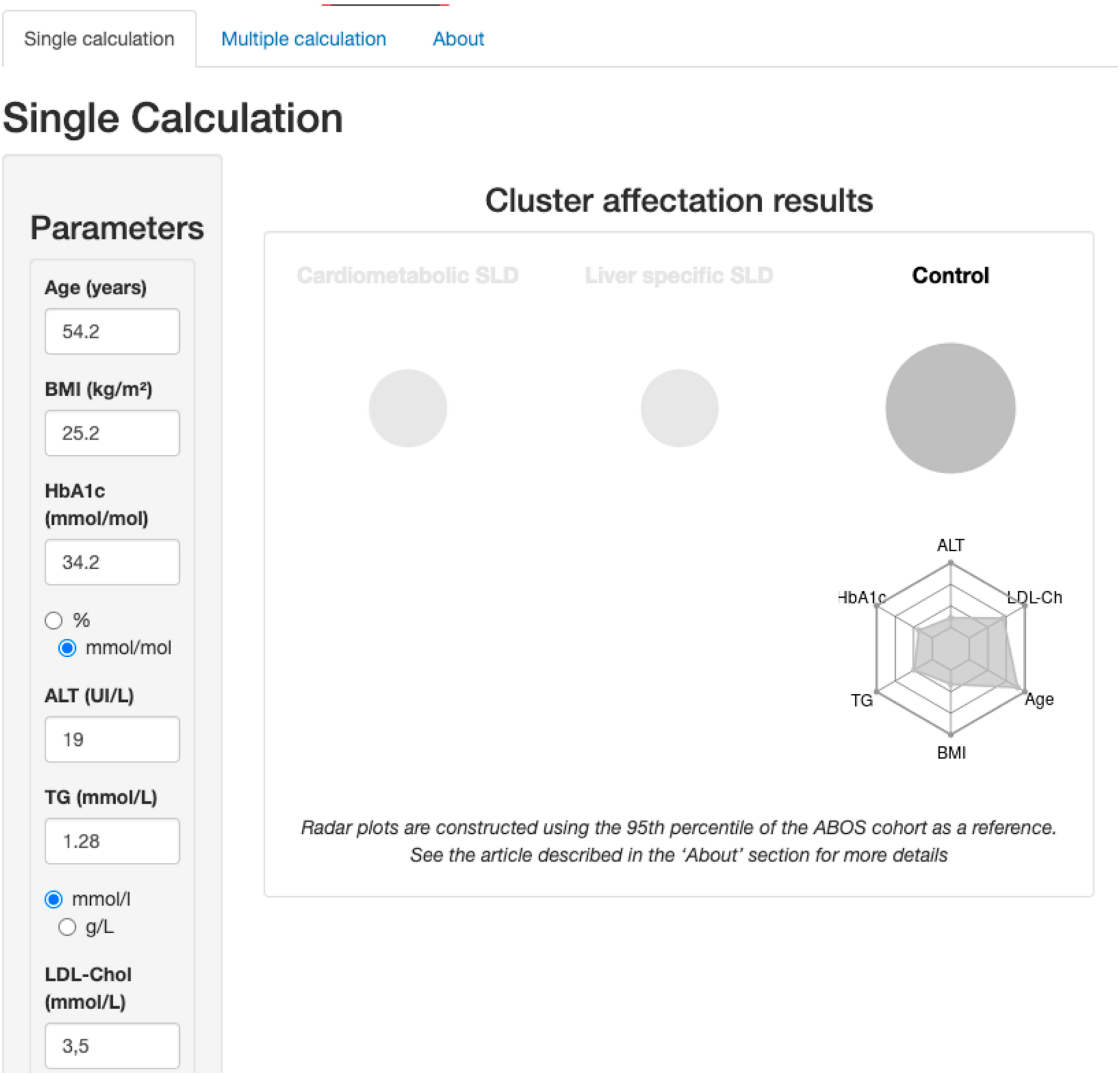

Supplement: Supplementary file 1 [file biomedicines-13-03045-s001.zip › S3 pheno cluster affectation.pdf]
